# Suppression for an intermediate phase in ZnSb films by NiO-doping

**DOI:** 10.1038/s41598-017-09338-3

**Published:** 2017-08-17

**Authors:** Chao Li, Guoxiang Wang, Dongfeng Qi, Daotian Shi, Xianghua Zhang, Hui Wang

**Affiliations:** 1 Laboratory of Infrared Material and Devices, The Research Institute of Advanced Technologies, Ningbo University, Ningbo, 315211 China; 2 Key Laboratory of Photoelectric Detection Materials and Devices of Zhejiang Province, Ningbo, 315211 China

## Abstract

The structural evolution and phase-change kinetics of NiO-doped ZnSb films are investigated. NiO-doped ZnSb films exhibit a single-step crystallization process, which is different from that of undoped ZnSb. NiO-doped ZnSb can directly crystallize into a stable ZnSb phase at temperatures greater than 320 °C with suppression of a metastable ZnSb phase. These characteristics enlarge the amorphous/crystalline resistance ratio by approximately five orders of magnitude. Moreover, NiO doping of ZnSb films increases crystallization temperature from 260 to 275 °C, improves data retention temperature from 201.7 to 217.3 °C and increases crystalline activation energy from 5.64 to 6.34 eV. The improvement of the thermal parameters in the nanocomposite can be attributed to stable ZnSb grain growth refinement owing to the dispersion of NiO particles in the sample matrix. This provides additional nucleation sites and produces more ZnSb/NiO interfaces, which can initiate the nucleation and accelerate crystallization. The kinetic exponent n decreases from 1.12 to 0.44, which confirms the ultrafast one-dimensional growth and heterogeneous phase transition of the NiO-doped ZnSb films. The improved thermal stability, larger resistance ratio and direct transition to a stable phase with ultrafast one-dimensional crystal growth indicate the good potential of these materials in phase-change memory applications.

## Introduction

On the basis of the resistivity change of phase-change materials, phase change memory (PCM) could be realized by a reversible transformation between amorphous and crystalline states induced by electrical pulses^1^. An amorphous high resistance state represents a binary ‘0’, while a low resistance crystalline state represents a ‘1’. Information can be stored in the phase of the material and read by measuring the resistance^2^. PCM has received considerable attention owing to its compatibility with existing complementary metal-oxide semiconductor (CMOS) technologies, remarkable read/write speeds (20 ns/10 ns), long cycle life (greater than 10^12^), and low environment influence^3^. However, the main limitation of PCM is the large power consumption of these devices. To reduce power consumption, researchers^4–7^ have searched for the phase-change materials with high crystalline resistance. It is well known that conventional Ge_2_Sb_2_Te_5_ (GST) alloy can crystallize in a metastable face-centered-cubic (fcc) structure upon heating crystallize (150 °C). At temperatures higher than 250 °C, the GST alloy can transform to a stable hexagonal close-packed (hcp) structure with a low crystalline resistance, which can lead to high power consumption in PCM applications^4^. Over the past few years, to suppress the fcc-to-hcp phase transition for PCM applications, great efforts have been made by doping small amounts of elements such as Zn^4^, C^5^, V^6^ and SnTe^7^ into conventional GST. However, the nucleation-dominated crystallization mechanism leads to slow crystallization.

Recently, Te-free compounds, including CSb^8^, GaSb^9^, GeSb^10^, ZnSb^11^, NSb^12^ and OSb^13^, have been widely used as phase-change materials because of their growth-dominated crystallization mechanism and rapid amorphous-to-crystalline transitions. Binary Zn-Sb systems, such as ZnSb and β-Zn_4_Sb_3_, are promising p-type materials^14^. In particular, ZnSb is a binary compound with a high carrier concentration of 10^19^ cm^−3^, which makes a major contribution to the change of the film resistance^14^. Moreover, compared with the properties of GST, ZnSb films present a high crystallization temperature (~257 °C), good data retention (~201 °C), low melting temperature (~500 °C), fast crystallization speed and high crystalline resistance^11^. Amorphous ZnSb films also exhibit a two-step crystallization process from an amorphous to a metastable ZnSb phase at 250 °C and then to a stable ZnSb phase at 350 °C^11^; however, the resistance ratio between the metastable and stable phases is limited to approximately one order of magnitude. This characteristic leads to facile phase separation and degradation of the reliability of the interface between the phase-change layer and the electrode in PCM devices. We previously attempted doping of elements, including Sn, In, and Al into ZnSb, for possible application as a phase-change layer^15^. We found that the properties of the ZnSb phase could be modified by the addition of the dopants; however, some critical issues remained, including the small resistance ratio of Zn-Sb-In^15^, the high temperature instability of Zn-Sb-Sn^15^ and phase separation (rhombohedral Sb + AlSb) of Zn-Sb-Al^15^. An innovative approach to addressing these issues is to use the nano-composite materials, incorporating dielectric and phase-change materials, to form an oxide/ZnSb interface, where no atomic migration or chemical reactions can occur^16^. These ZnSb-based nano-composite films are a new kind of phase-change material, and their high crystalline resistance is expected to enable reduced power consumption in PCM applications.

In this paper, we report the local structure and phase-change kinetics of nano-composite NiO-doped ZnSb films for PCM applications. X-ray diffraction, *in situ* resistance measurements and Raman spectra reveal that NiO-doped ZnSb films can directly crystallize into a stable ZnSb phase with suppression of the intermediate metastable ZnSb phase. The modified crystallization process, induced by NiO-doping can address the phase separation problem, improve thermal stability, and enlarge the resistance ratio of ZnSb films. Atomic force microscopy and transmission electron microscopy images indicate that NiO-doped ZnSb films can provide additional nucleation sites and new ZnSb/NiO interfaces. Moreover, the NiO-doped ZnSb materials can maintain growth-dominated characteristics and possess different growth modes, as confirmed from analysis based on fundamental nucleation and growth theories.

## Results and Discussion

**Figure 1:**
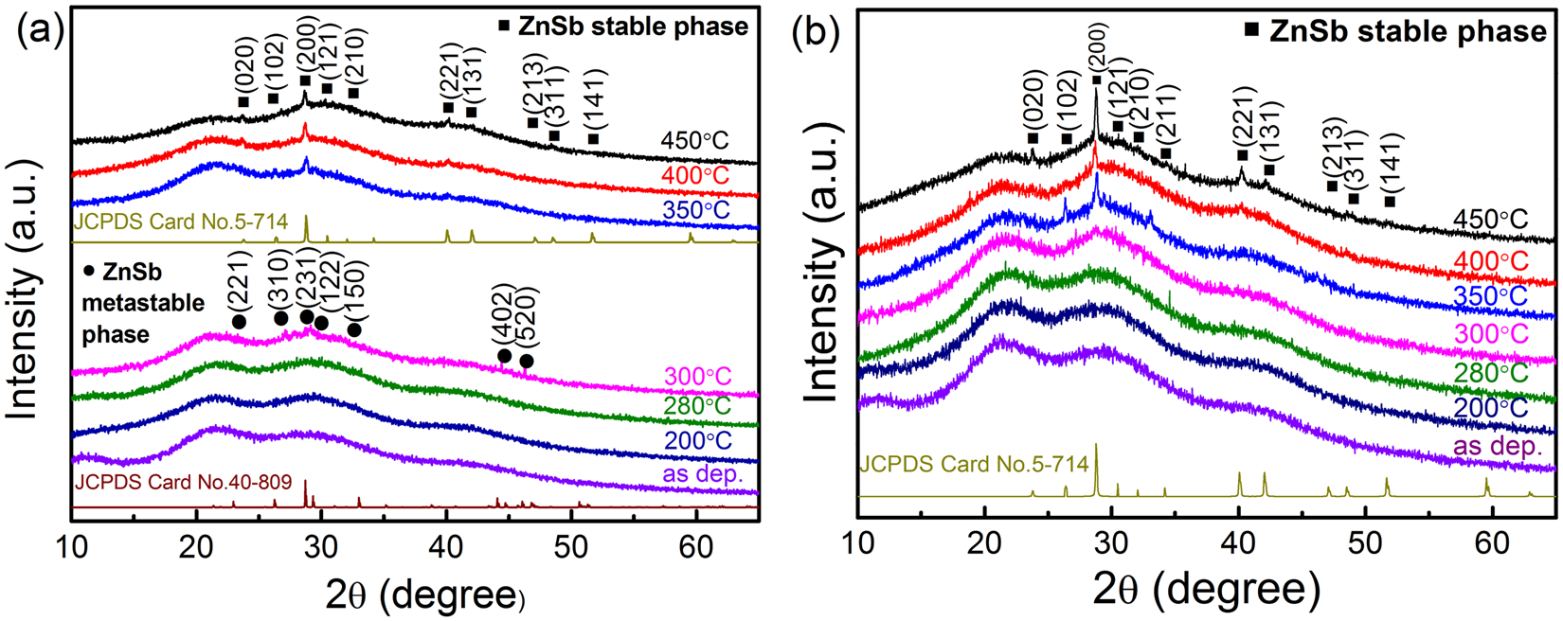
XRD patterns of as-deposited and annealed (**a**) undoped and (**b**) NiO-doped ZnSb films. At the bottom are shown the peak positions and intensity taken from metastable and stable ZnSb phase in the XRD database for comparison.

Figure 1 shows the XRD patterns of 70 nm-thickness ZnSb and ZnSb-NiO films annealed at different temperatures for 10 min, respectively. As shown in Fig. 1(a), no sharp diffraction peaks are observed in the as-deposited ZnSb film. The amorphous phase is remained stable up to an annealing temperature of 280 °C. A set of diffraction patterns corresponding to a metastable ZnSb phase appears in the ZnSb film after annealing at 300 °C. As the annealing temperature is increased from 350 to 450 °C, a phase transformation from a metastable ZnSb phase to a stable ZnSb phase occurs. The investigated ZnSb film exhibits a two-step crystallization process: an amorphous → metastable ZnSb transition at 300 °C and a metastable ZnSb → stable ZnSb transition at 350 °C. The corresponding phases could be indexed in the XRD database as JCPDS no. 40–809 and JCPDS no. 5–714, respectively. Although the thin 70 nm ZnSb film exhibits a similar two-step crystallization process to that of the 120 nm ZnSb film^11^, the crystallization temperature for the transition from the amorphous to the metastable ZnSb phase increases. This is mainly attributed to the constrained sample size for grain growth during recrystallization^17^^.^Heat transfer through the sample becomes more difficult as the film thickness decreases. The restrictions on energy supply might also delay the phase transition^18^. This effect can also be attributed to the increased specific interface energies and inhomogeneous strain that occur for the thinner film, as elucidated from the Zacharias’ model^19^. This model considers the specific interfacial energy that interpolates among the interface energies of the oxide, amorphous and crystalline phases. It is assumed that a crystalline nucleus forms in the center of the amorphous layer and grows towards the boundaries of the oxide. For a thin film, the effect of the additional specific interfacial energy increases the nucleation barrier and the difficulty of nucleation.

The XRD patterns of NiO-doped ZnSb films as shown in Fig. 1(b) exhibit a different crystallization behavior. The amorphous phase remains until 300 °C and then begins to crystallize at 350 °C. The annealing temperature increases from 400 to 450 °C. At this temperature, the film achieves full crystallization and a stable ZnSb phase precipitates. The addition of NiO suppressed the amorphous-to-metastable ZnSb phase transition. Notably, the (102) peak could be observed at 350 °C, but is gradually suppressed at 400 and 450 °C. The other crystallization peaks also become much sharper. These results might be attributed to a change in the preferred orientation of the crystalline phase.

**Figure 2:**
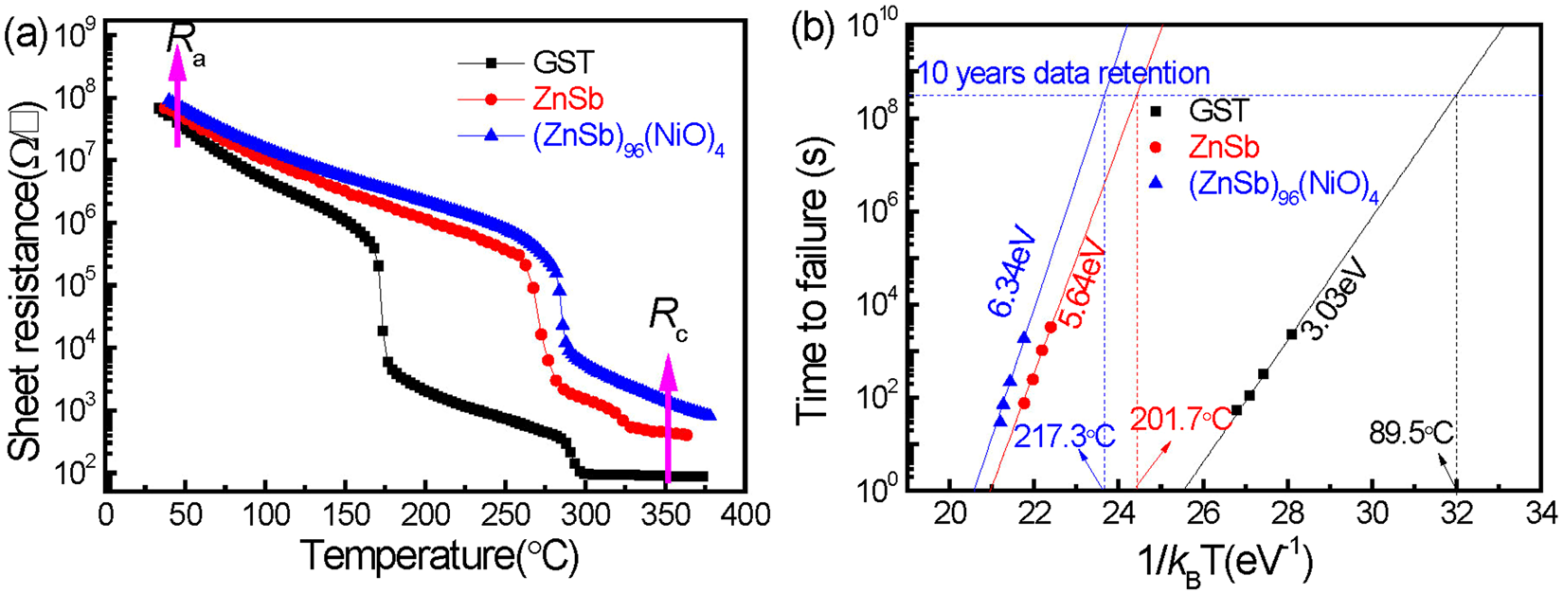
(**a**) Sheet resistance as a function of temperature for GST, undoped and NiO-doped ZnSb films. (**b**) The Arrhenius extrapolation at 10-year of data retention for GST, undoped and NiO-doped ZnSb films.

Figure 2 shows the variation of sheet resistance as a function of increasing temperature (R–T curve) at a heating rate of 40 °C/min for GST, ZnSb and NiO-doped ZnSb films. For all samples, the sheet resistance decreases gradually with increasing temperature. However, a sudden drop occurs when the temperature reaches the respective crystallization temperature (*T*_c_), which corresponds to the minimum of the first derivative of R-T curve. *T*_c_ values are determined to 260 and 275 °C for undoped ZnSb and NiO-doped ZnSb films, respectively. Both these values are higher than that of conventional GST films (168 °C). The increment of *T*_c_ could be attributed to the restriction of surface atomic motion on the ZnSb nanocrystals, which is caused by coherent bonding with surrounding atoms at the interfaces^20^. Notably, the GST film exhibits another transition at 280 °C, which corresponds to a fcc-to-hcp phase transition. Similarly, a slight transition in the ZnSb films at 320 °C corresponds to a metastable ZnSb-to-stable ZnSb phase transition, implying that the ZnSb alloy crystallizes in a metastable state and then transforms to a stable structure at high temperature. Unlike ZnSb, the NiO-doped ZnSb films exhibit no second resistance drop at high temperature and crystallize directly in the stable phase. According to TEM images, the disappearance of the intermediate state (metastable phase) between the amorphous and stable phase can be attributed to accelerated crystallization of the stable phase, which results from incorporation of a highly-resistive NiO phase and the presence of ZnSb/NiO interfaces. Thus, the amorphous/crystalline resistance (*R*_a_/*R*_c_) ratio is increased from ~5.7 × 10^4^ to ~1.1 × 10^5^ and the thermal stability is also improved.

Figure 2(b) presents the results of crystalline activation energy (*E*_a_) and the maximum temperature for 10-year data retention (*T*_10-year_), which further demonstrates the thermal stability of the amorphous phase. These parameters are calculated by the Arrhenius equation^16^: $t=\tau \exp ({E}_{a}/{k}_{B}T)$, where *t* is the time to failure, *τ* is a proportional time constant, *E*_a_ is the activation energy, *k*_B_ is the Boltzmann constant, and *T* is the thermodynamic temperature. The failure time *t* is defined as the time at which the sheet resistance decreases to half of its initial value at the specific temperature *T*. As shown in Fig. 2(b), the *T*_10-year_ value of the amorphous (ZnSb)_96_(NiO)_4_ film is determined to be 217.3 °C with an *E*_a_ of 6.34 eV. These results are much higher than those of GST (89.5 °C, 3.03 eV) and ZnSb (201.7 °C, 5.64 eV), indicating that the addition of NiO increases the *T*_10year_ and *E*_a_ values. This enhancement can be attributed to refinement of the ZnSb grains and the dispersion of NiO particles in the sample matrix, which interrupt the lattice periodicity. Lattice discontinuities, such as grain boundaries in ZnSb and the newly formed ZnSb/NiO interfaces, retard phonon propagation and thus reduce thermal conduction. This mechanism can be inferred from the increase in the thermal resistance (*R*_a_, *R*_c_) of the nanocomposite, as shown in Fig. 2(a). The NiO-doped ZnSb films with a higher thermal resistance limit heat propagation, which contributes to the increase of *T*_10-year_ and *E*_a_. On the basis of the above analysis, these parameters, including the *T*_c_, *R*_a_/*R*_c_, *E*_a_ and *T*_10-year_ values, represent an improvement over those of metal Zn-, Al-, In-doped ZnSb films^15^.

**Figure 3:**
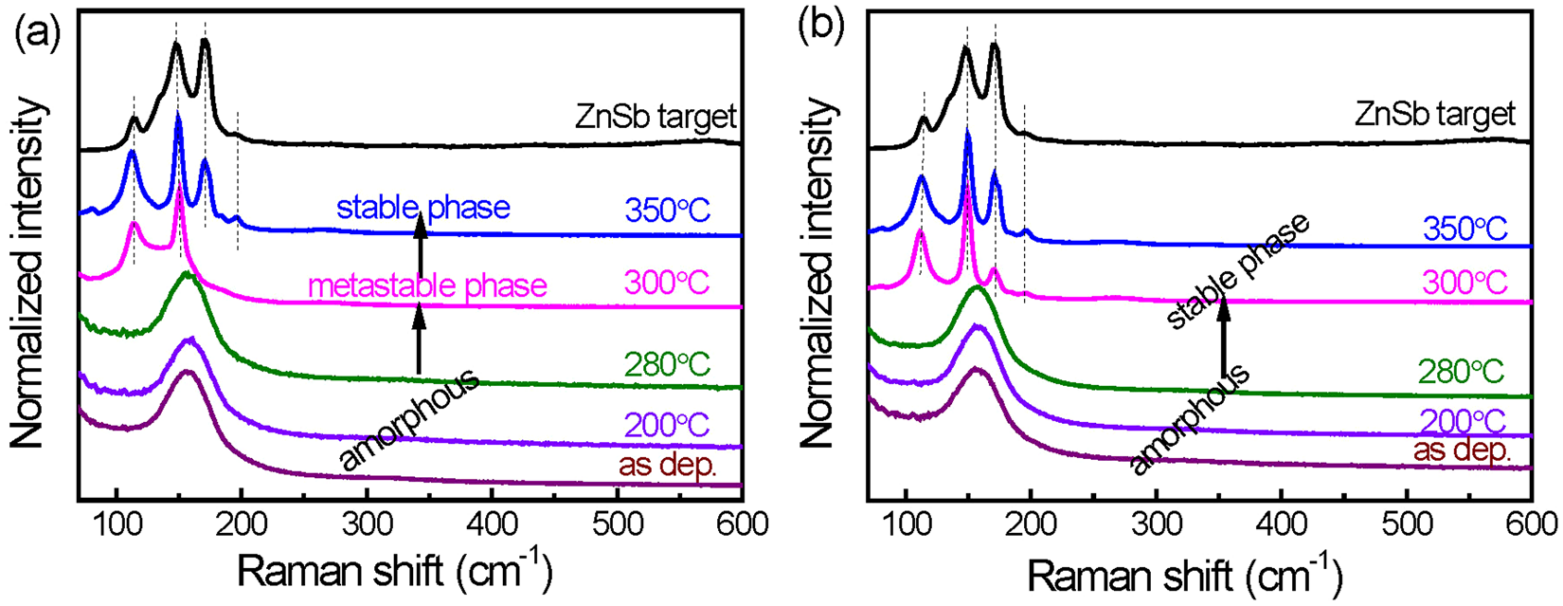
Raman spectra of as-deposited and annealed (**a**) undoped and (**b**) NiO-doped ZnSb films.

The phase transition is further examined by Raman analysis. Figure 3 shows the Raman spectra of undoped ZnSb and NiO-doped ZnSb films annealed at different temperatures for 10 min, respectively. As shown in Fig. 3(a), the broad Raman peak of the as-deposited, 200 °C- and 280 °C-annealed ZnSb films implies the presence of an amorphous phase. Two vibrational peaks observed in the 300 °C-annealed film correspond to a metastable ZnSb phase. Subsequently, the transformation from a metastable to a stable phase occurs at 350 °C. For comparison, the Raman peaks of the NiO-doped ZnSb films are shown in Fig. 3(b). These spectra show a direct change from a broad peak to several vibrational peaks, which are assigned to the stable ZnSb phase. The annealing temperature for this transition is increased from 200 to 350 °C. Notably, there are no NiO vibrational peaks observed at 560 cm^−1^ ^21^, and no crystallization peak of NiO (111) in the XRD patterns^21^_._ This result confirms that NiO presents as an amorphous phase and acts a center for suppressing the intermediate phase.

**Figure 4:**
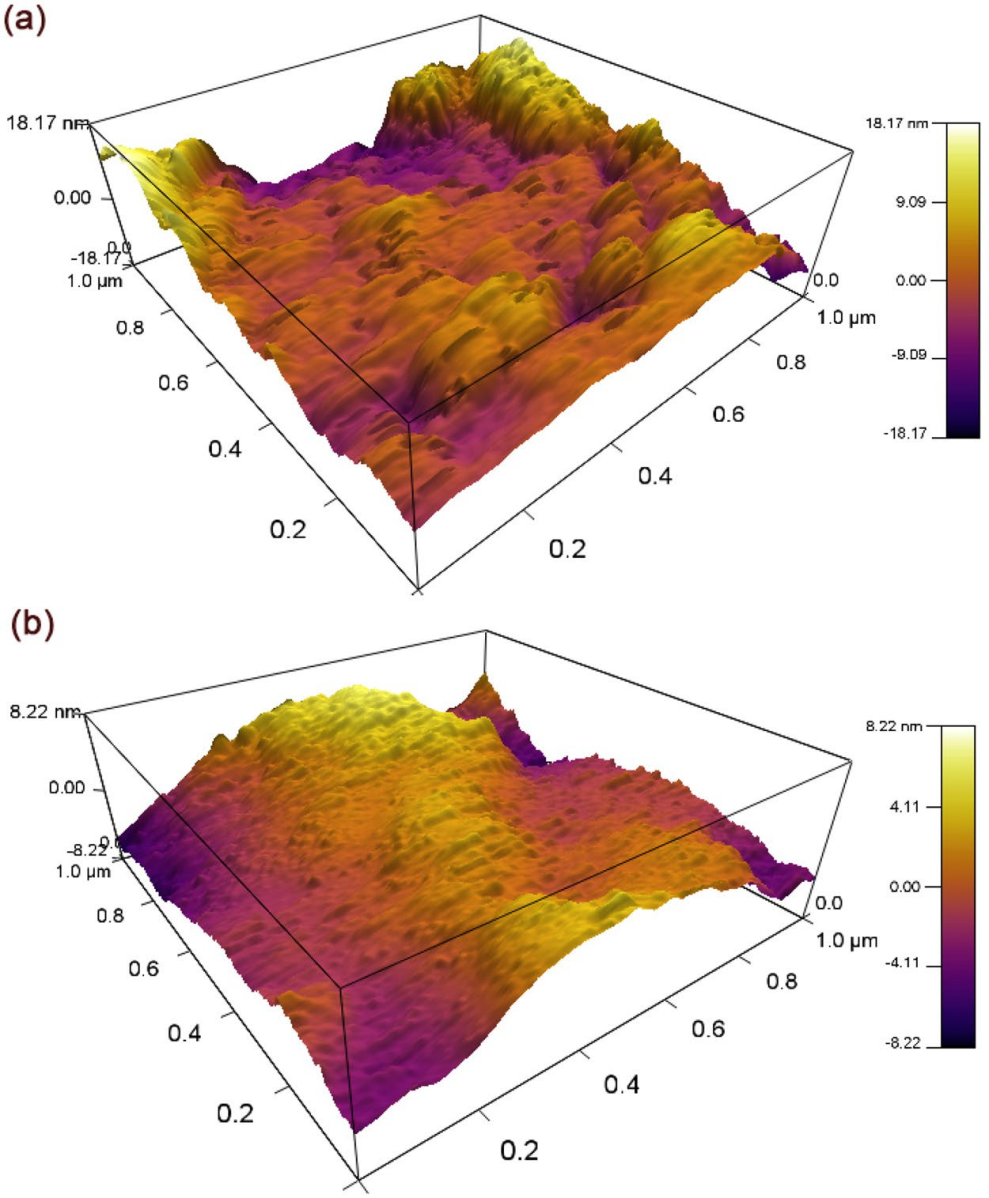
AFM images of 350 °C-annealed (**a**) ZnSb and (**b**) (ZnSb)_96_(NiO)_4_ as a three-dimensional images with the same scale.

The surface morphology reflects changes in the nucleation density and growth modes; hence, we investigated the morphological changes using AFM, as shown in Fig. 4. The scan area in Fig. 4(a,b) is 1 µm × 1 µm. Compared with the AFM image of the ZnSb film as shown in Fig. 4(a), the grain size is reduced in the NiO-doped ZnSb films as shown in Fig. 4(b). The number of large grains gradually decreases and more nuclei appear in the NiO-doped ZnSb films. Thus, the root mean square (RMS) values are 8.964 and 4.855 nm for the undoped and NiO-doped ZnSb films, respectively. This result implies that the addition of NiO can improve the surface quality of the ZnSb films.

**Figure 5:**
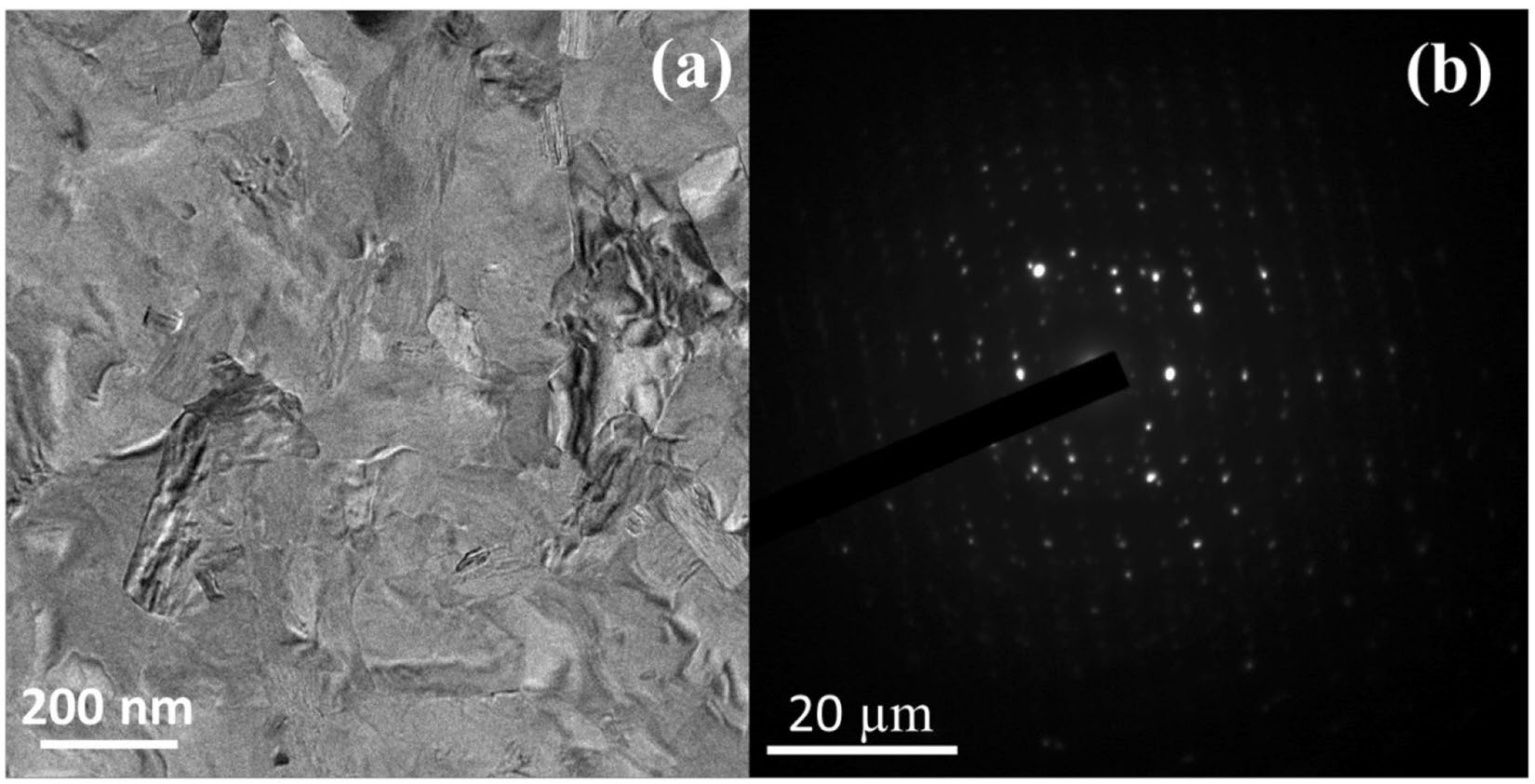
(**a**) The bright field TEM image and (**b**) the SAED pattern of the undoped ZnSb film annealed at 350 °C.

Figure 5 shows TEM micrographs and corresponding selected area electron diffraction (SAED) patterns for the undoped ZnSb films annealed at 350 °C. Many large crystals appear to be distributed in TEM image as shown in Fig. 5(a) with fringes and bend contours, together with discrete diffraction dots in the SAED pattern as shown in Fig. 5(b). These features are characteristics of a single-crystal^22^.

**Figure 6:**
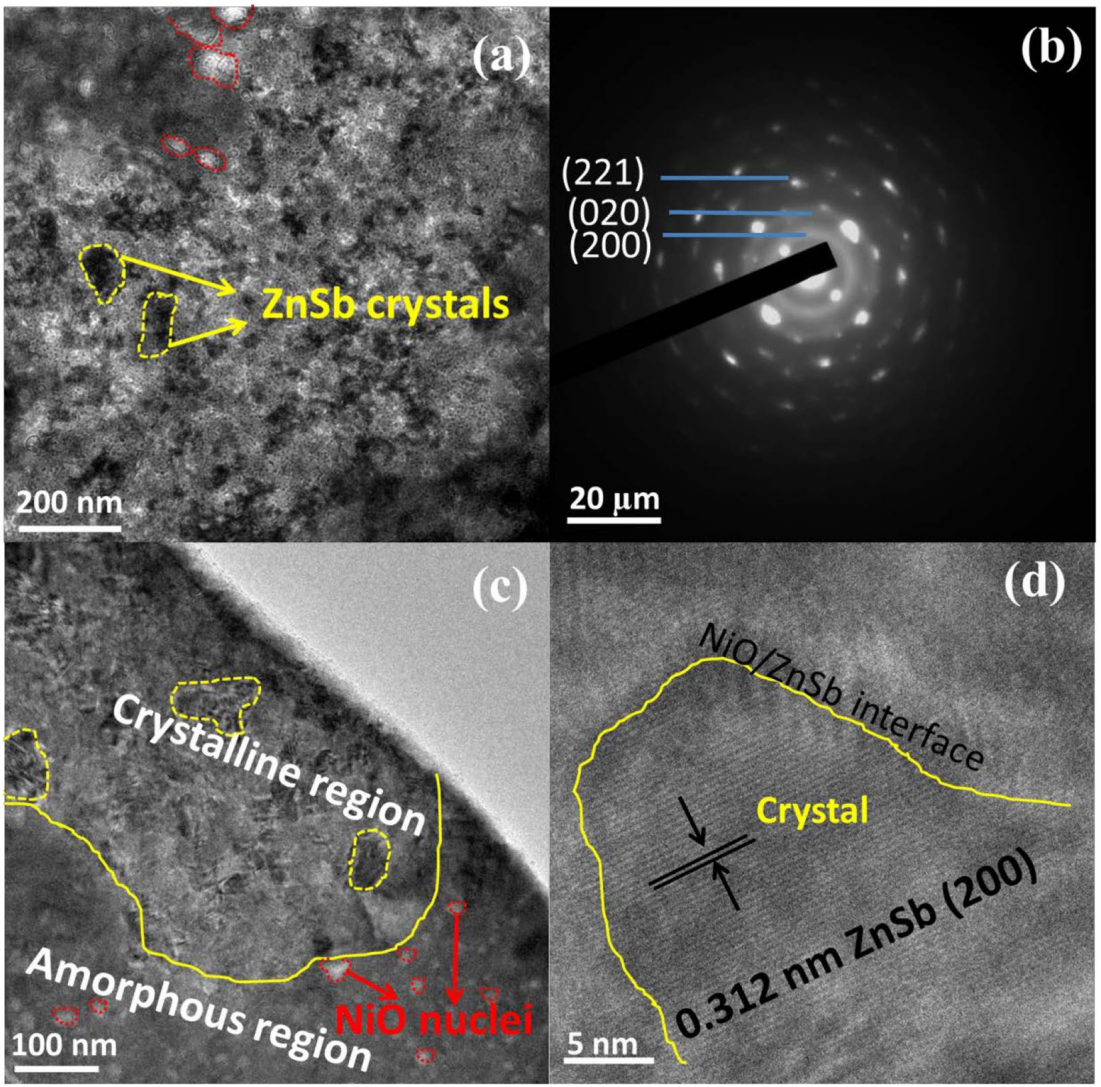
(**a**) The bright field TEM image, (**b**) the SAED pattern (**c**) the cross-section bright filed TEM image and (**d**) the HRTEM image of the NiO-doped ZnSb film annealed at 350 °C.

The addition of NiO suppresses the crystal growth in the NiO-ZnSb film as shown in Fig. 6(a). It is found that the annealed film presents an uniform phase morphology, embedded with bright and dark regions corresponding to an amorphous phase and ZnSb crystal grains, respectively. The crystalline grain size is considerably smaller than that of the ZnSb film. The number of small grains markedly increases with the addition of NiO, which is consistent with our observations of the AFM morphology in the crystalline NiO-doped ZnSb. SAED patterns as shown in Fig. 6(b) suggests that the diffraction rings are related to diffraction from a polycrystalline structure, which is confirmed to be the stable ZnSb phase. Clear amorphous and crystalline regions are also found in a cross-sectional TEM image as shown in Fig. 6(c), which confirms that the film crystallizes as a nanocomposite with crystalline ZnSb grains surrounded by an amorphous phase. In the amorphous region, more NiO crystalline nuclei are formed in the center of the amorphous material and grow towards the ZnSb/NiO interfaces. Notably, no phase separation occurs in the NiO-ZnSb alloy after annealing at 350 °C, confirming our analysis of the XRD and Raman spectra results. In high-resolution transmission electron microscopy (HRTEM) image, a stable ZnSb phase with a (200)-lattice spacing of 0.312 nm appears and a NiO/ZnSb interface is formed, which serves as a preferential site for nucleation and affects the crystalline growth modes of NiO-ZnSb films.

**Figure 7:**
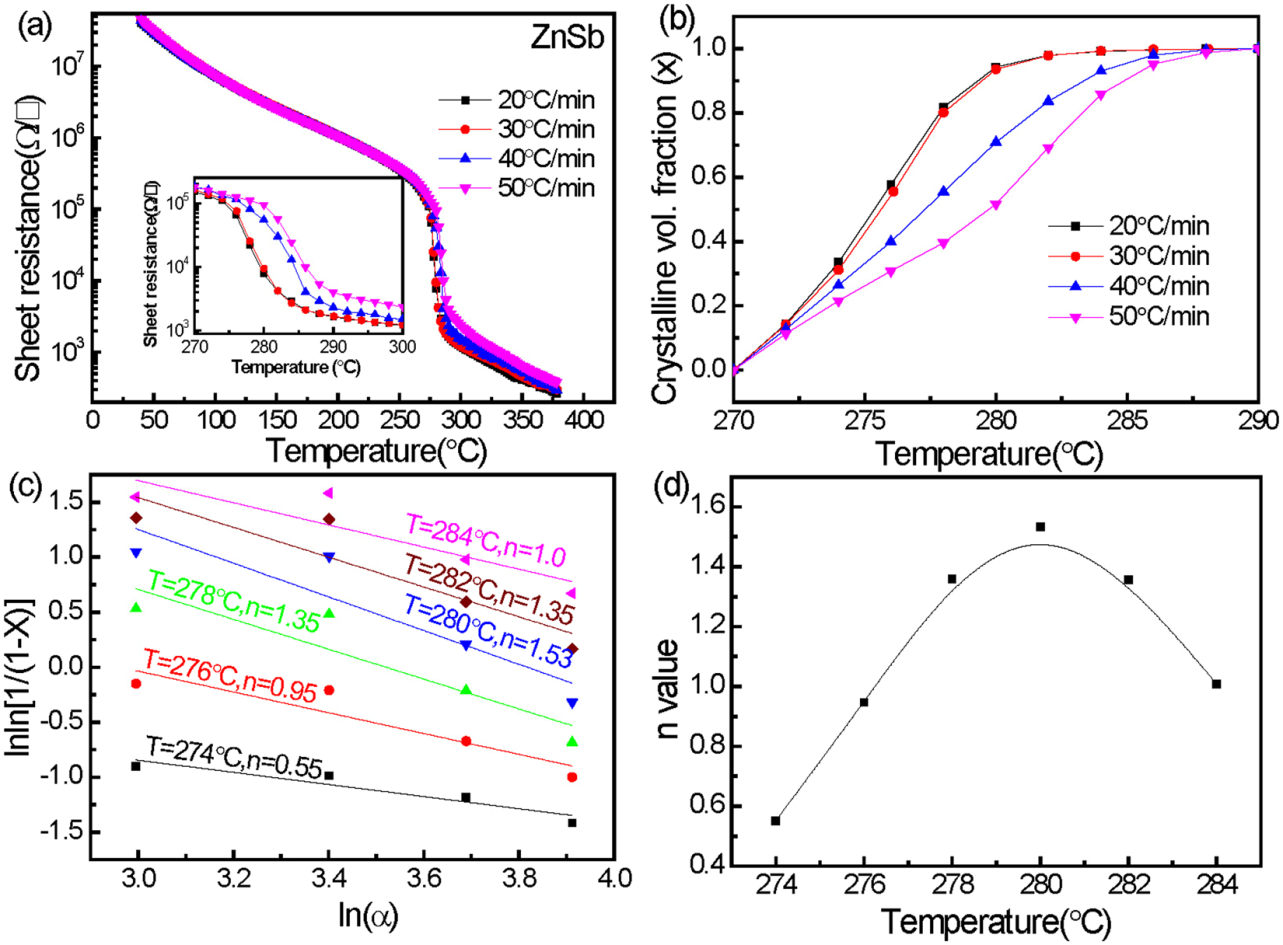
Results taken from ZnSb film using the of (**a**) R–T curves at different heating rates, (**b**) curves showing fraction of crystallization at different heating rates, (**c**) Ozawa’s plot, and (**d**) temperature-dependent kinetic exponents.

**Figure 8:**
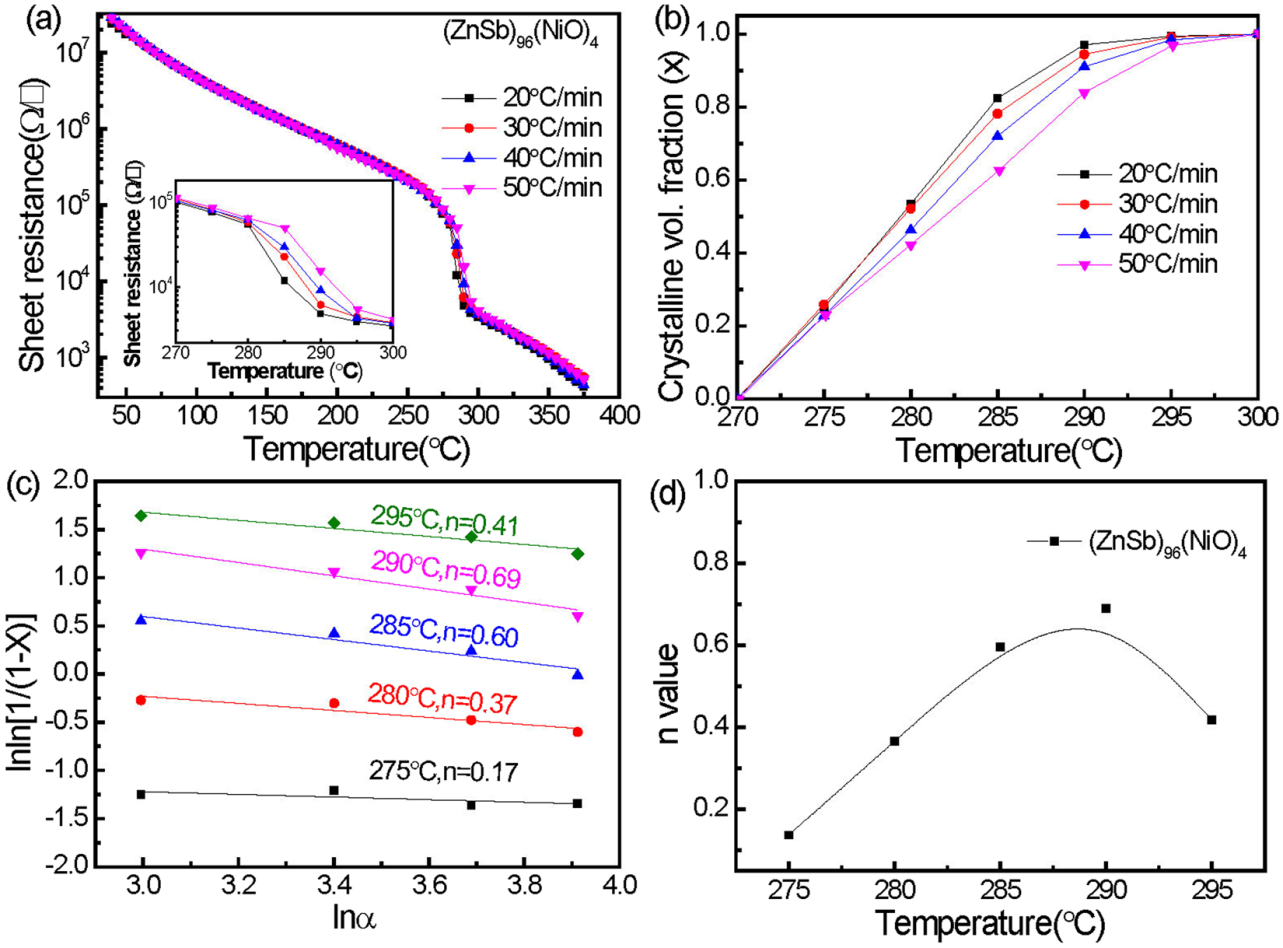
Results taken from (ZnSb)_96_(NiO)_4_ film using the of (**a**) R–T curves at different heating rates, (**b**) curves showing fraction of crystallization at different heating rates, (**c**) Ozawa’s plot, and (**d**) temperature-dependent kinetic exponents.

The crystallization mechanism of the ZnSb and NiO-ZnSb films was investigated further as shown in Figs 7 and 8, respectively. The R-T curves of the ZnSb and (ZnSb)_96_(NiO)_4_ films at several different heating rates (α) were measured as shown in Figs 7(a) and 8(a), respectively. The inset shows an enlargement of the transition over a temperature range of 270 to 300 °C. The derivative of the curves versus temperature is determined in this temperature range. By integrating the derivatives against temperature and normalizing, transformation curves reflecting the crystallized fraction can be obtained. The crystallization fraction (x), defined as the ratio of the integrated area of the crystallized part to that of the whole, is plotted as a function of temperature as shown in Figs 7(b) and 8(b). On the basis of the Ozawa model, including the kinetic exponent n^23^, linear fitting of the relation between ln[ln(1/(1−x))] and lnα at several different temperatures allows for determination of n as shown in Figs 7(c) and 8(c). The kinetic exponent n as a function of temperature is shown in Figs 7(d) and 8(d). Different n values correspond to different crystallization mechanisms, which are normally categorized as growth-dominated when n is less than 1.5 and nucleation-dominated when n is greater than 1.5^24^. According to Figs 7(d) and 8(d), as the annealing temperature increases, the n value first increases and then decreases. Owing to the different temperatures, deviation of the n values from the mean value is found for each composition. Notably, both the mean n values are lower than 1.5, implying one-dimensional crystal growth from nuclei. The mean n value (0.44) of the (ZnSb)_96_(NiO)_4_ is much smaller than that of ZnSb (1.12), indicating that the incubation period for crystallization is relatively short. The decrease of the Avrami exponent (n) indicates that the phase transition is likely to be heterogeneous^25^, because the dispersed NiO particles provide additional nucleation sites. These nuclei can accelerate the crystalline growth in an ultrafast one-dimensional mode.

## Conclusions

The effects of NiO doping on the phase change properties of ZnSb have been studied. *In situ* sheet resistance measurements in conjunction with XRD and Raman analysis reveal that NiO-doped ZnSb transforms from an amorphous phase to a stable ZnSb phase. The addition of NiO increases the crystallization temperature, 10-year data retention temperature and crystalline activation energy. TEM characterizations confirm that embedded NiO provides more nuclei and that ZnSb/NiO interfaces can refine the grain growth and accelerate crystallization in nanocomposite films. Compared with ZnSb, the crystallization mechanism of (ZnSb)_96_(NiO)_4_ features a lower *n* value of 0.44, indicating faster one-dimensional crystal growth from the nuclei. The direct transition to a stable phase, large resistance ratio, good thermal stability and one-dimensional crystallization growth mode indicate that the (ZnSb)_96_(NiO)_4_ film is an excellent candidate for PCM applications.

## Methods

### Sample preparation

The 70 nm-thickness NiO-doped ZnSb film was deposited on quartz and SiO_2_/Si (100) substrates by a magnetron co-sputtering method using individual ZnSb and NiO targets. The size of the quartz and SiO_2_/Si substrates were both 2 cm × 2 cm, with RMS roughness of less than 0.8 and 0.5 nm, respectively. The substrate temperature was kept at room temperature. In each run of the experiment, the base and working pressures were set to be 5.6 × 10^−4^ and 0.3 Pa, respectively. The Ar gas flow was set to 47.6 mL/min. The direct current power (*P*_dc_) and the radio frequency power (*P*_rf_) was fixed at 15 and 50 W, applied to a NiO target of 50 mm diameter and the ZnSb target of 50 mm diameter, respectively. Undoped ZnSb and GST films with the same thickness were also prepared for comparison.

### Characterizations

The thickness of the film was measured by surface profiler (Veeco Dektak 150). The stoichiometry of the as-deposited films was confirmed by X-ray photoelectron spectroscopy (XPS, AXIS UltraDLD, Japan). The concentration of NiO in the ZnSb film was determined to be around 4 atomic % (at%). The sheet resistance of the as-deposited films was measured *in situ* using a four point probe in a vacuum chamber built in-house, as a function of temperature (non-isothermal) or time (isothermal). The structure of as-deposited and annealed films was examined by X-ray diffraction (XRD, D2 Phaser, Bruker, Germany) and Raman spectra (InVia, Renishaw, UK). The diffraction patterns were taken in the 2θ range of 10–60° using Cu Kα radiation with a wavelength of 0.154 nm and performed under Bragg conditions for samples. The Raman scattering spectroscopy was recorded at room temperature using a backscattering configuration and an Ar ion laser with a wavelength of 785 nm as the excitation source. The power density incident on the sample was kept at low levels (~0.2 mW µm^−2^) to avoid laser-induced structural modification and the resolution of the Raman spectra is 1 cm^−1^. The morphological differences between undoped ZnSb and NiO-doped ZnSb films were observed to investigate the relationship between the nucleation center and the mechanism of the fast phase change speed using atomic force microscopy (AFM). The microstructure of the films was observed using transmission electron microscopy (TEM).

## References

[1] Krebs D, Bachmann T, Jonnalagadda P, Dellmann L, Raoux S (2014). Changes in electrical transport and density of states of phase change materials upon resistance drift. New J. Phys..

[2] Rios C (2015). Intergated all-photonic non-volatile multi-level memory. Nat. Photonics..

[3] Zhou X (2016). Phase Change Memory materials by design: a strain engineering approach. Adv. Mater..

[4] Wang GX (2012). Phase change behaviors of Zn-doped Ge_2_Sb_2_Te_5_ films. Appl. Phys. Lett..

[5] Zhou X (2014). Understanding phase-change behaviors of carbon-doped Ge_2_Sb_2_Te_5_ for phase-change memory application. ACS Appl. Mater. Interfaces..

[6] Zhang T, Zhang B, Shao RW, Zheng K (2014). Structural evolution and corresponding electrical properties of V-doped Ge_2_Sb_2_Te_5_ with increased temperature. Mater. Lett..

[7] Xu JA (2012). High speed phase change memory based on SnTe-doped Ge_2_Sb_2_Te_5_ material. Electrochem. Solid-State Lett..

[8] Chang CC (2011). Phase stability, bonding and electrical conduction of amorphous carbon-added Sb films. Scripta Mater..

[9] Putero M (2014). Density change upon crystallization of Ga-Sb films. Appl. Phys. Lett..

[10] Eising G, Damme TV, Kooi BJ (2014). Unraveling crystal growth in GeSb phase-change films in between the glass-transition and melting temperatures. Cryst. Growth Des..

[11] Chen YM (2014). Crystallization behaviors of Zn_x_Sb_100−x_ thin films for ultralong data retention phase change memory applications. CrystEngComm..

[12] Hu Y (2016). Improved thermal stability of N-doped Sb materials for high-speed phase change memory application. Appl. Phys. Lett..

[13] Hu Y (2017). O-doped Sb materials for improved thermal stability and high-speed phase change memory application. J. Alloy Compd..

[14] Zheng ZH (2015). The influence of the transformation of electronic structure and micro-structure on improving the thermoelectric properties of zinc antimonide thin films. Intermetallics.

[15] Chen YM (2016). The feasibility of Sn, In, or Al doped ZnSb thin film as candidates for phase change material. J. Appl. Phys..

[16] Wuttig M, Steimer C (2007). Phase change materials: From material science to novel storage devices. Appl. Phys. A..

[17] Christian, J. W. The theory of transformations in metals and alloys, part I, equilibrium and general kinetictheory, 2nd ed., pp.15, 525, Pergamon, Oxford (1975).

[18] Huang YJ, Chung TC, Wang CH, Hsieh TE (2010). Characterizations of AgInSbTe and its nanocomposite Thin Films for Phase-change memory applications. J. Electrochem. Soc..

[19] Zacharias M, Streitenberger P (2000). Crystallization of amorphous superlattices in the limit of ultrathin films with oxide interfaces. Phys. Rev. B.

[20] Ohshima N (1998). Structural analysis and crystallization studies of germanium–antimony–tellurium sputtered films on different underlayers. J. Appl. Phys..

[21] Kakehi Y, Nakao S, Satoh K, Kusaka T (2002). Room-temperature epitaxial growth of NiO(111) thin films by pulsed laser deposition. J. Cryst. Growth..

[22] Zhou X (2011). Sb-rich Si-Sb-Te phase change material for multilevel data storage: The degree of disorder in the crystalline state. Appl. Phys. Lett..

[23] Ozawa T (1971). Kinetics of non-isothermal crystallization. Polymer.

[24] Cheng HY, Kao KF, Lee CM, Chin TS (2008). Crystallization kinetics of Ga–Sb–Te films for phase change memory. Thin Solid Films..

[25] Jang MH (2015). Ultrafast phase change and long durability of BN-incorporated GeSbTe. J. Mater. Chem. C..

